# Remodelling and Improvements in Organoid Technology to Study Liver Carcinogenesis in a Dish

**DOI:** 10.1155/2019/3831213

**Published:** 2019-02-19

**Authors:** Umesh Tharehalli, Michael Svinarenko, André Lechel

**Affiliations:** Department of Internal Medicine I, Ulm University, Ulm, Germany

## Abstract

Primary liver cancer (PLC) is the sixth most common tumour disease and one of the leading causes of cancer-related death worldwide. The two most common types of PLC are hepatocellular carcinoma (HCC) and intrahepatic cholangiocarcinoma (iCCA). Diverse subgroups are described and a manifold number of gene mutations are known. Asymptomatic disease progression and limited therapeutic options are the reasons for the high mortality rate in PLC. Up to date, the multikinase inhibitors sorafenib and lenvatinib are the only FDA-approved first-line treatments for advanced HCC. One of the major drawbacks in the preclinical drug development is the lack of suitable model systems. In recent years, 3D organoid cultures were established from several organs and tumour subtypes, thereby opening new avenues in tumour research. 3D organoid cultures are used to describe the tumour diversity, for cancer modelling in a dish and for therapy responsiveness. The establishment of living biobanks and the development of next-generation matrices are promising approaches to overcome drug resistance and to improve the quality of personalised anticancer strategies for patients with PLC. In this review, we summarise the current knowledge of 3D cultures generated from healthy liver and primary liver cancer.

## 1. Worldwide Importance of Primary Liver Cancer and the Need for Therapies

Primary liver cancer (PLC) is the second most common cause of cancer-related death worldwide and has an incidence of over 800,000 new cases per year [1, 2]. Liver cancer belongs to the neoplasms with increasing incidence and mortality rates [2, 3]. Moreover, liver cancer consists of a heterogeneous group of different malignant tumour entities with distinct histological features and poor prognosis rates. Hepatocellular carcinoma (HCC) represents with roughly 80% the majority of all PLC followed by intrahepatic cholangiocarcinoma (iCCA) [4]. Other neoplasms of PLC which account for less than 1% are mixed hepatocellular cholangiocarcinoma (HCC-iCCA), fibrolamellar HCC (FLC), and hepatoblastoma (usually affecting young children) [5, 6]. Risk factors for liver cancer are well known. The main risk factors associated with HCC are viral hepatitis (HBV/HCV), alcohol abuse, nonalcoholic fatty liver diseases (metabolic syndrome, diabetes), and aflatoxin B1 exposure. Viral hepatitis is also described as a risk factor associated with iCCA; others are primary sclerosing cholangitis, biliary duct cysts, hepatolithiasis, and parasitic biliary infestation with liver flukes which are a major risk factor in Southeast Asia [7]. In contrast to HCC, the majority of patients suffering from iCCA were not exposed to any risk factor [8].

Most patients with PLC are diagnosed at advanced stages, often when a tumour is already metastasized. Several conventional chemotherapies have been tested, but with a limited survival benefit [9]. So far, the multikinase inhibitors sorafenib and lenvatinib are the only FDA-approved first-line treatments against HCC [10, 11]. Additional targeted drugs failed to meet clinical endpoints in phase III trials, except the sorafenib derivate regorafenib and the immune checkpoint inhibitor nivolumab which showed potency in second-line treatments for advanced HCC [12-14].

## 2. Organoids as a Promising *In Vitro* Model System

A major problem in the preclinical drug development is the diversity of etiologies and the broad variety in the genetic landscape of PLC [15-20]. Currently, we are lacking good model systems to understand the histopathology of liver cancer. Since decades, a lot of research was performed on the basis of 2D cultures and transgenic mouse models [21, 22]. Cell lines established from primary liver tumours can be grown as a 2D culture, but there are several pitfalls exist in using 2D cell lines. The high diversity of existing etiologies and the broad range of the genetic landscape of liver tumours indicated by sequencing studies cannot be represented by the limited number of available cell lines. Problems often occur by taking tumours into the culture, next to contamination risks of the primary material, far not all tumours give rise to primary cells. Especially, cells from HCC tumours with mutations like Wnt/*β*-catenin are nearly impossible to cultivate up to date. Moreover, through the process of evolutionary selection, only a certain clone with the most beneficial mutation will expand under *in vitro* culture conditions. This results in a loss of the tumour heterogeneity and the survival of the fittest clone.

2D cell lines fail to mimic the histoarchitecture and do not fully represent the genomic landscape of PLC. To achieve a better model which mimics the histoarchitecture and the genomic landscape of liver cancer, researchers succeeded in establishing culture conditions for an organoid system. Organoids are characterized by a self-organizing 3D structure which mimics the original *in vivo* architecture of organs or tumours and can be derived from different sources. So far, organoids could be derived from organ-specific adult stem cells, from pluripotent stem cells, which can be either embryonic stem cells or induced pluripotent stem cells and from tumours (called as tumouroids) [23-25].

**Figure 1:**
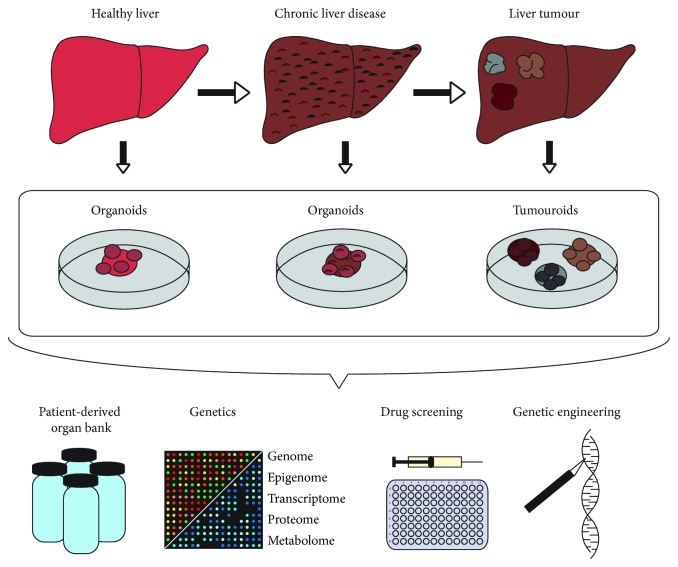
Establishment of organoids from healthy liver tissue, liver with chronic disease, and liver carcinoma and their downstream applications.

Organoids are established for the use of multiple applications. One of the most promising applications is to build up a biobank of patient-derived organoids/tumouroids resembling the heterogeneity of individual tumour entities. This database could be used for a personalised therapy based on the results achieved from organoids/tumouroids drug screening. For this purpose, organoids/tumouroids will undergo genetic profiling (Figure 1).

Mutational analysis and also data from the transcriptome, the epigenome, the proteome, and the metabolome can be achieved. After that, organoids/tumouroids can be used for a mutation-specific or a pathway-directed drug testing. The results could be collected in a commonly available database. Therapeutic strategies could be transferred in future to medicate cancer patients who carry the same mutations. Apart from cancer, organoids are also used for liver disease modelling which can be targeted by gene editing to modulate specific mutations. Another interesting application of organoids is to study host-microbe interactions. Intestinal organoids provide a model system to examine the response of the epithelium to the presence of enteric pathogens and the symbiosis of the host intestinal surface and the gut microbiota [26].

## 3. Establishment of 3D Organoid Cultures from the Adult Liver

Modelling human liver diseases *in vitro* is a challenging task. Primary hepatocytes (PH) have a limited proliferative capacity *in vitro*; they dedifferentiate fast during culture and lose their viability and function upon cryopreservation [27-29]. Therefore, the establishment of efficient methods to cultivate PH over a larger time span is in urgent need to understand disease mechanisms and for the use of therapeutic implications. Liver-derived organoids are a great tool for modelling diseases in a dish. They enable genetic modifications and drug screening, to improve the quality of personalised medicine and cell transplantation therapy.

Interesting approaches for the long-term 3D cell culturing techniques were proposed in the late 1990s. The technology consists of a bioreactor and beads coated with an extracellular matrix (ECM) for cell attachment. Khaoustov and colleagues successfully established long-term propagation of a 3D system for liver cells which were also called organoids but which are different from the recently established 3D organoids. The experimental setup consists of the isolation of liver cells including hepatocytes, cholangiocytes, stellate cells, and sinusoidal endothelial cells and the use of scaffolds made of polyglycolic acids [30]. These scaffolds are porous in nature and biocompatible, making a convenient atmosphere for the attachment of liver cells. Liver cells are incubated with scaffold pouches (bilayer scaffold) and subsequently transferred to bioreactors to generate 3D organoids. Interestingly, this technique allows liver cells to assemble into an organ-like manner, while the hepatocytes are functional with the secretion of albumin and other liver-specific enzymes [31]. Several studies use this bioreactor technology to compare the 2D cell culture and the 3D organoid system. Chang and Hughes-Fulford isolated PH from mice and plated them simultaneously in monolayer tissue culture dishes (2D) and in the rotating wall vessel (RWV, 3D) bioreactors. Although PH in 2D culture had a growth advantage, they underwent epithelial-mesenchymal transition following cell culture. In contrast, the 3D RWV bioreactor allows PH to form organoids with improved hepatocyte-specific functions, which are defined by higher albumin production and increased CYP1A1 activity along with retention of epithelial markers [32]. Moreover, 3D organoids show upregulated gene expression of *Hnf4α* and its downstream targets, which have been described as master regulators of functional hepatocytes [32, 33]. The bioreactor technique is advantageous for exploring the mechanism of cell-cell communication and mixed-cell culture techniques but is nontransferable for *in vivo* applications and thereby differs from recently developed 3D organoid systems [32, 34, 35].

**Figure 2:**
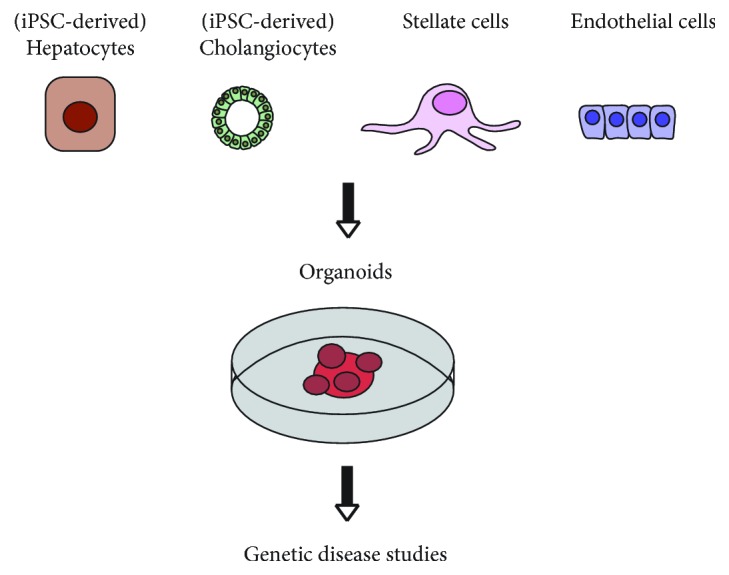
iPSC-derived mixed-cell liver organoids for disease modelling.

Several years have elapsed since the generation of liver organoids was established from PH and biliary epithelial cells (BEC) [36]. Huch and colleagues established liver organoids from EpCAM- PH and EpCAM+ BEC isolated from human donor liver tissue [37]. Interestingly, only EpCAM+ BEC successfully transform into 3D organoids. Addition of forskolin (FSK) and removal of R-spondin (Rspo-1) from the specific isolation medium allow ductal organoids to undergo hepatocyte differentiation. Further, FGF19 and dexamethasone were added in the specific expansion medium to enable differentiated hepatocytes to produce bile acids and to show glucose and lipid metabolism to make them functionally active. Further, the addition of BMP7 accelerates hepatocyte proliferation and simultaneously allows differentiated cells to acquire hepatocyte morphology [36]. Broutier and colleagues adopted and modified this procedure; some of the critical steps are summarised in Table 1. These liver organoids serve as a bridge between patients and transgenic animal models in understanding chronic liver disease and could be used for drug testing [38–40]. Apart from primary epithelial cells and stem cells, induced pluripotent stem cells (iPSCs) serve as a tool for the establishment of tissue-specific organoids (Figure 2) [36, 37, 41, 42].

Till date, transgenic animals serve as model systems to explore rare diseases, yet they fail to recapitulate disease traits. Modulating disease traits in transgenic animals is time-consuming and requires a good understanding of the disease. In addition to transgenic animal models, we need a fast tool to simulate the disease and explore treatment modalities. *In vitro* approaches, especially 3D culture techniques, serve as an advantage over the animal models. The study from Takebe et al. [41] and Guan et al. [42] uses iPSC to generate liver organoids. Due to the fact that the liver is a heterogeneous organ, the organoids were generated by combining iPSC-derived liver cells (iPSC-LC) with stromal cells and endothelial cells. The process of obtaining liver organoids from human iPSC includes differentiation of iPSC to endodermal spheres (ES) and then culture on a low concentration Matrigel (1%-2%) to promote formation of posterior foregut-like structure (PFS). Further, addition of certain growth factors to PFS promotes lineage-specific differentiation and maturation towards hepatocytes and cholangiocytes (Table 1) [42]. The successful transplantation of *in vitro*-derived organoids would require the establishment of vasculatures to achieve a metabolic functionality. The approach from Takebe and colleagues combines iPSC-LC with mesenchymal stem cells and endothelial cells (HUVEC). Interestingly, the cells self-organise to form a 3D appearance in the coculture. *In vivo* transplantation experiments reveal that endothelial cells are essential to establish vascular structures. Within 48 hours of transplantation, they establish vascular connections with the host liver, supported by mesenchymal stem cells from the transplantation. With the advanced gene editing technology, these organoids can be genetically manipulated to generate a disease-specific model with known mutations or to improve future cell transplantation therapies [41, 43].

## 4. Organoids from Liver Carcinoma

Protocols from the establishment of primary liver cell-derived 3D organoids were adopted and modified (Table 1) to generate organoids from primary liver tumours described as tumouroids [37]. Primary liver tumours are heterogeneous consisting of epithelial cells, cancer-associated fibroblasts, and other nonepithelial cells. Hence, primary liver tumours were digested between a few hours to overnight at 37°C (30 minutes to 1 hour for liver tissue) to improve the yield of tumour cells and to minimize nonepithelial cell contamination. Tumour cells were then plated with the basement membrane extract (BME). BME mainly provides stability and maintains a differentiated state of the epithelial cells. For long-term propagation of tumouroids, Broutier and colleagues implied two cultivation mediums. One-half of the tumouroids were maintained in the classical human organoid isolation medium (IsoMed) [37] and the second half in the tumouroid-specific medium (TSM) [38]. The TSM consists of IsoMed [37] without the factors promoting Wnt/*β*-catenin and BMP4 signalling (R-spondin-1, noggin, and Wnt3a condition medium).

### 4.1. HCC Tumouroids

HCC tumours are associated with cellular and molecular heterogeneity, chromosomal aberrations, and both somatic and germ line mutations. Nuciforo and colleagues established HCC tumouroids from a patient-derived needle biopsy and compared it with the corresponding tumour biopsy. Most of the HCC tumouroids resemble original tumour biopsy in terms of growth pattern, differentiation grade, expression of HCC-specific markers, and ability to form tumours in xenograft models. Additionally, HCC tumouroids retain genetic mutations [38, 40] as well as chromosomal aberrations [38] but some of the HCC tumouroids gain additional mutations in the culture [40]. The success rate of establishing HCC tumouroids from the tumour biopsy depends on the proliferation rate of tumour cells and differentiation stage of the liver tumours and is not affected by the clinicopathological condition of the patient [38, 40].

### 4.2. iCCA Tumouroids

Like HCC tumouroids, iCCA-derived tumouroids represent most of the features of the corresponding tumour tissue biopsy. Interestingly, the mutational spectrum of iCCA tumours is diverse, meaning that a particular set of mutations is exhibited only in a small subgroup of cells within the tumour. Though iCCA tumouroids retain most of the driver gene mutations, some of the tumouroids lost somatic mutations in culture. iCCA tumouroids recapitulate most of the iCCA tumour tissue phenotypes such as the solid/trabecular growth, the presence of atypical cells, the production of mucin, and the presence of intracytoplasmic lumen structures [38, 40].

## 5. Organoids Derived from PLC as a Tool for Drug Screening

PLC is often diagnosed at late stages due to the lack of prognostic markers. Though tyrosine kinase inhibitors (sorafenib, lenvatinib, and regorafenib) provide a survival benefit, the response rate is limited to a small subgroup of patients [10, 11, 13]. Tools and database for deciding suitable drugs for therapies are therefore in urgent need. The human PLC-derived tumouroids recapitulate most of the histological features including tumour-specific secretory phenotype, mutational landscape, and chromosomal aberrations of the corresponding tissue biopsy. Hence, organoids/tumouroids are a reliable tool for testing drug toxicity, sensitivity, and efficacy to make patient-specific therapeutic choices. The sensitivity of several anticancer drugs was attributed to the tumour classification and mutational signature of the tumours [39]. Therefore, the high tumouroid reproducibility of the mutational landscape of the primary tumour allows for a large-scale screening for anticancerous compounds and their sensitivity. This approach could help to establish a more promising medication strategy. In a multicompound testing, Broutier and colleagues could show to what extent drug sensitivity is linked to specific mutations. For example, a patient-derived HCC tumouroid with *CTNNB1* mutation was resistant to Wnt inhibitors, whereas a Wnt-dependent iCCA tumouroid was sensitive to the same inhibitor [38]. In contrast, the same HCC tumouroid displayed sensitivity to EGFR inhibitors, whereas the *KRAS*-mutated iCCA tumouroid was resistant. Moreover, the drug screening on tumouroids revealed sorafenib, which is only approved for HCCs, as a promising medication also for certain iCCAs [38, 40]. Interestingly, for successful treatment, it is important to choose not only the right pathway but also the right member. For instance, tumouroid lines across all entities showed resistance against BRAF and MEK inhibitors, but were sensitive towards inhibitors of the downstream target ERK1/2 [38]. These findings could be also validated in xenograft models. Hence, generating a database which consists of tumour type, driver mutations, and sensitivity/resistance of a particular drug would help to make fast decisions on using suitable chemotherapeutics.

## 6. Next-Generation Designer Matrices

The generation of tumour-derived organoids is a great achievement. However, so far, aspects such as the tumour microenvironment cannot be addressed simultaneously with the 3D tumouroid culture. The microenvironment of the primary liver tumour consists of stromal cells, blood vessels, and a variety of immune cells. Culture conditions may affect the natural arising heterogeneity of the tumour and might be beneficial for certain subpopulations within the tumour. A key component of the organoid culture is the Matrigel, which is a heterogeneous complex mixture of extracellular matrix proteins (e.g., proteoglycans) and growth factors secreted by the murine sarcoma cell line Engelbreth-Holm-Swarm. Matrigel is poorly defined in its composition; it is not mechanically pliable after plating and depicts compositional and structural variation from lot-to-lot which links to its limited clinical translational potential [44, 45]. The undefined and variable composition of the Matrigel might have pathogenic, immunogenic, and metastatic side effects [45]. Recently, polyethylene glycol-based hydrogel synthetic formulations are also described as next-generation matrices used for intestinal stem cell and organoid cultures [45-48]. The aim was to create a minimal, chemically defined environment by using enzymatically crosslinked polyethylene glycol (PEG) hydrogels [49]. For the design of mechanically dynamic matrices, a hybrid PEG hydrogel was created to decrease the stiffness. Therefore, the stable polymer backbone (sPEG, mechanically static PEG) was mixed with a hydrolytically degradable polymer (dPEG, mechanically dynamic PEG) [45, 50]. Furthermore, a RGD (Arg-Gly-Asp) peptide and laminin-111 were added to change the stiffness and made it accessible for organoid formation [45]. A completely synthetic PEG-based hydrogel was presented by Cruz-Acuña and colleagues for the expansion of human intestinal organoids [51, 52]. Their formulation consists of a four-arm PEG macromere with maleimide groups at each terminus (PEG-4MAL) which mimics minimal toxicity and inflammation *in vivo* [53, 54]. Moreover, PEG-4MAL exhibits a high cytocompatibility [51]. First reports show that designer matrices are used for the cultivation of organoids from liver and liver tumour [55, 56]. Fibrin-based and liver-derived ECM-based hydrogels were used for the culture of organoids derived from the human liver [55, 56]. In addition, HCC patient-derived xenograft (PDX) organoids derived from HCC-PDX lines were cultured in a 3D macroporous hydrogel. This macroporous cellulosic sponge system consists of a hydroxypropyl cellulose (HPC) which is grafted with methacrylic groups (MA) to render the polymer photo-crosslinkable [57, 58]. The availability of different designer matrices and their exchangeable components is an ongoing process, which definitely leads to greater experimental possibilities for organoid culture systems.

## 7. The Living Biobank

Precision oncology is a clinical approach where a custom strategy for cancer patients is designed based on the genetic profile of an individual tumour. The methodology of next-generation sequencing results in huge data collections of the genomic landscape of several cancer types. These data sets were collected in consortia like The Cancer Genome Atlas (TCGA) or the International Cancer Genome Consortium (ICGC). The generation and integration of data from human tumouroids and organoids belong to one of the major achievements in the field of oncology. The use of a living biobank generated from human tumouroids or PDXs, together with genomic profiling and high-throughput drug screenings, helps to improve clinical decisions.

Over the last five years, the generation of human tumouroids has been described for prostate cancer [59, 60], ductal pancreatic cancer [61], colorectal cancer [62], breast cancer [63], bladder cancer [64], gastric cancer [65], and liver cancer [38, 40]. The generation of a living biobank has been started for most of these cancers followed by a drug screening for the potential future therapeutic strategies.

Tumouroids could be generated from surgically resected PLC tissue resembling the main types: HCC, iCCA, and a combined type called hepatocellular cholangiocarcinoma [38]. In addition, tumouroids could be achieved from tumour needle biopsies of HCC patients with various etiologies and tumour stages [40]. Those tumouroids could be cultivated as long-term cultures while retaining the histopathological characteristics and expression pattern of HCC markers, thereby maintaining the genetic heterogeneity of the primary tumour. In the same study, tumouroids could be generated from poorly differentiated cholangiocarcinoma which displayed similar histological properties like the original tumour. A few cells within the tumouroid displayed intracytoplasmic lumens and produced mucins which are characteristic features of adenocarcinomas [40].

Several reports suggest successful long-term cultivation of tumouroids derived from organ-specific tumours. These tumouroids retain their morphologies that typically match the histopathology of the corresponding primary tumour. The spectrum of genetic changes within the living biobank correlates or remains closely similar to the primary tumour. However, we should be aware of possible changes which can occur during long-term cultivation. The matrices used may not always generate the same optimal culture condition for the whole tumour cell population and may not lead to a representation of the whole intratumoural heterogeneity.

## Figures and Tables

**Table 1:** Comparison of isolation and culture conditions for organoids and tumouroids.

Liver tissue-derived organoids	Liver tumour-derived organoids	iPSC-derived organoids
Liver tissue was digested for up to 1 hour in the digestion medium (DM).	Liver tumours were digested in DM between 2 hours to overnight depending the degree of fibrosis. Longer digestion helps with reducing contaminating ductal cells.	(i) At first, iPSCs are dissociated into single cells and allowed to undergo into endoderm spheres (ES) in a special endoderm differentiation medium. (ii) These ES are then cultured on a low concentration (1%-2%) Matrigel to facilitate the formation of posterior foregut-like structure (PFS). (iii) By treating with various concentration of FGF10, PFS obtain hepatic and gallbladder cell morphology (HG). (iv) Addition of chemicals such as hepatic growth factor (HGF), oncostatin, and dexamethasone on a low concentration Matrigel (1%) promotes differentiation of HG cells into hepatic organoids (HOs). (v) Since HOs are limited in proliferation and regeneration upon reaching a size of 3 mm in diameter, HOs were further dissociated and cultivated to promote proliferation and differentiation separately.
Digestion was stopped once the tissue was completely dissociated, following centrifugation at 300-400g; cell pellets were washed in cold Advanced DMEM/F12 medium. Cells were then mixed with BME2 and seeded.		
Further on, the liver organoids were then maintained in “classical human organoid isolation medium” (IsoMed) for 1 week in culture.	One-half of the tumouroids were maintained in IsoMed and the other half was maintained in “tumouroid-specific isolation medium” TSM (IsoMed without noggin, Rspo-1, and Wnt3a condition medium) in order to ensure the growth of the cultures.	
	Tumouroids were maintained in their corresponding isolation medium until the first split (approximately 2-3 weeks).	
For long-term expansion, these organoids and tumouroids were then cultured in human healthy liver-derived expansion medium (IsoMed without Wnt3a condition medium, noggin, and Y27632).		
